# HIV-1 tropism: a comparison between RNA and proviral DNA in routine clinical samples from Chilean patients

**DOI:** 10.1186/1743-422X-10-318

**Published:** 2013-10-28

**Authors:** Pablo Ferrer, Luisa Montecinos, Mario Tello, Rocio Tordecilla, Consuelo Rodríguez, Marcela Ferrés, Carlos M Pérez, Carlos Beltrán, Maria A Guzmán, Alejandro Afani

**Affiliations:** 1 Laboratorio de Medicina Molecular, Hospital Clínico Universidad de Chile, Santiago, Chile; 2 Laboratorio de Infectología y Virología Molecular, División de Pediatría, Escuela de Medicina, Pontificia Universidad Católica de Chile, Santiago, Chile; 3 Centro de Biotecnología Acuícola, Facultad de Química y Biología, Universidad de Santiago de Chile, Santiago, Chile; 4 Departamento de Infectología, Complejo Asistencial Barros Luco. Facultad de Medicina, Universidad de Santiago de Chile, and Chilean AIDS Cohort (ChiAC), Santiago, Chile

**Keywords:** HIV proviral DNA, HIV coreceptor, CCR5, CXCR4, Genotypic tropism test

## Abstract

**Background:**

HIV in Chile has a notification rate of 0.01%. Coreceptor antagonists are a family of antiretroviral drugs that are used with the prior knowledge of patients HIV-1 tropism. Viral RNA-based tropism detection requires a plasma viral load ≥1000 copies/mL, while proviral DNA-based detection can be performed regardless of plasma viral load. This test is useful in patients with low or undetectable viral loads and would benefit with a proper therapy. The aim of this study was to determine the correlation between HIV RNA and proviral genotypic DNA tropism tests.

**Findings:**

Forty three Chilean patients were examined using population-based V3 sequencing, and a geno2pheno false-positive rate (FPR) cutoff values of 5, 5.75, 10 and 20%. With cutoff 5.75% a concordance of 88.4% in tropism prediction was found after a simultaneous comparison between HIV tropism assessment by RNA and DNA. In total, five discrepancies (11.6%) were found, 3 patients were RNA-R5/DNA-X4 and two were RNA-X4/DNA-R5. Proviral DNA enabled the prediction of tropism in patients with a low or undetectable viral load. For cutoff 5 and 5.75% genotypic testing using proviral DNA showed a similar sensitivity for X4 as RNA. We found that the highest sensitivity for detecting the X4 strain occurred with proviral DNA and cutoff of 10 and 20%. Viral loads were higher among X4 strain carriers than among R5 strain carriers (p < 0.05).

**Conclusions:**

A high degree of concordance was found between tropism testing with RNA and testing with proviral DNA. Our results suggest that proviral DNA-based genotypic tropism testing is a useful option for patients with low or undetectable viral load who require a different therapy.

## Findings

Currently there are 26,740 notified HIV cases in Chile and there is an estimate of 30 new confirmed cases every week [1]. New classes of antiretroviral drugs have been developed to control HIV infection among which are CCR5 coreceptor inhibitors. However, their use requires a prior tropism test to assess the type of coreceptor used by the virus and are generally phenotypic [2]. These tests are very expensive and difficult to perform, thus being incompatible with routine diagnostic procedures. For this reason, genotypic viral tropism assays using viral RNA have been developed [3]. However, RNA-based genotypic testing is generally restricted to patients with viral loads ≥1000 copies/mL, thus its use in patients with low or undetectable viral loads is limited [2]. To overcome this issue, DNA-based testing has been explored, supported by the idea that proviral DNA is the genetic archive containing all previous mutations of the virus [4]. In fact, several articles about HIV tropism recommend the use of proviral DNA for prediction of HIV tropism in patients with low o undetectable viral load. The concordance between RNA and proviral DNA test range between 74 and 97.6%, depending of the type and subtype of HIV [5-7]. According to the European Guidelines the determination of HIV tropism must be determined in each population and country and is particularly relevant in drug-naive patients, with toxic effects or for whom antiretroviral therapy (ART) has failed and a change in treatment is considered [8]. HIV tropism for Chilean patients under ART and virologic failure has not been reported and it is unknown if the virologic failure is associated to a particular HIV tropism. We addressed this issue testing HIV tropism using viral RNA and proviral DNA simultaneously in 43 patients belonging to the Chilean AIDS Cohort [9]**.** These patients did not have previous determination of viral tropism nor treatment with Maraviroc. Patients were selected according to the following inclusion criterion: under ART and having at least one virologic failure. This work was approved by the Ethics Committee of the Hospital Clínico Universidad de Chile. Table 1 shows the epidemiological and clinical characteristics of this group. In addition, 50 samples were analyzed to estimate the prevalence of R5 and X4 strains among Chilean patients. This group of patients underwent the same inclusion criterion and their epidemiological and clinical features were similar to the first group (Additional files 1: Table S1 and 2: Table S2).

**Table 1:** **Patient**^**# **^**epidemiological and clinical fetures (n = 43)**

Age (Range)	45 (18:70)*
Gender (Male;Female)	(34:9)
CD4 count (Cells/mm^3^)	232 (5;1162)*
Viral load (Log RNA copies/mL)	3.94 (3.08;5.70)*

*Median and Range.

# All patients with HIV clade B.

Viral RNA was extracted from plasma with EasyMag (Biomerieux). V3 loop of HIV-1 was amplified by One step RT PCR was performed in triplicate for each sample then cDNA was used as template for a nested PCR. Total DNA was extracted from whole blood using QIAamp® DNA Mini Kit (Qiagen). Nested PCR to amplify V3 loop of HIV-1 in Proviral DNA also was performed in triplicate [10]. Each PCR product was sequenced with the Sanger’s traditional sequencing method using the 3730xl DNA Analyzer (Applied Biosystems®) in Macrogen Company (USA). Sequences were analyzed using RECall [11,12]. The approved sequences were used to predict tropism using geno2pheno (G2P) and a false-positive rate (FPR) cutoff of 5%, 5.75%, 10% and 20% (Additional file 3: Table S3) [3,10,13]. We choose G2P over PSSM (Position-specific scoring matrices) due to that G2P and PSSM have equal percentage of concordance for subtype B, the most common in Chile, but G2P has been more used in clinical routine analysis in the prediction of HIV tropism [14]. Three predictions were obtained for each sample and the lowest FPR was considered for the overall prediction of viral tropism. Statistical analysis of data was carried out with non-parametric testing in the SigmaPlot V10 Software. Viral loads were determined with Nuclisens Easy Q v2.0 (Biomerieux). CD4 lymphocyte counts were performed with BD TruCount™ (Becton Dickinson).

**Figure 1:**
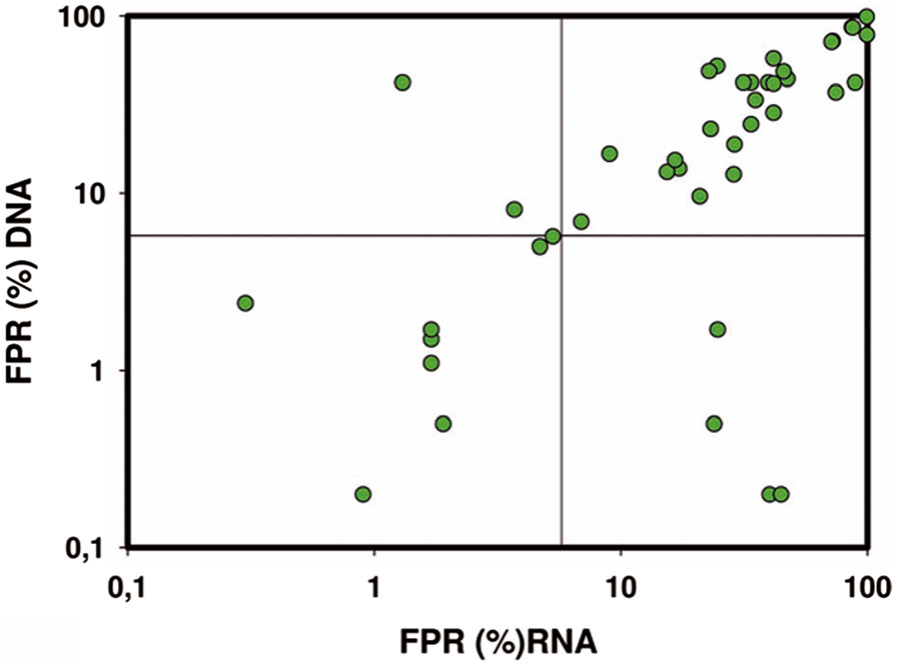
**Dispersion graph for simultaneous comparison between results of RNA–based prediction of tropism (x axis) and proviral DNA–based prediction (y axis) for 43 paired RNA/DNA samples.** Predictions were performed with the geno2pheno software and results were expressed as false positive rate (FPR;%). The correlation coefficient was ρ = 0.817 (P = 2.39 × 10^-11^).

For each sample, the lowest FPR obtained through RNA was correlated with the lowest FPR obtained through proviral DNA (Figure 1). The correlation coefficient (ρ) between both determinations was 0.817 with a p value of 2.39 × 10^-11^. The concordance in prediction between RNA and proviral DNA was 88.4%. Such results are similar to those found in previous articles [5-7].

When analyzing RNA, 74.4% of patients had R5 tropism and 25.6% had X4 tropism. For proviral DNA, 72.1% showed a R5 tropism and 27.9% had X4 tropism. The percentage of patients with the same tropism either determinated by RNA or proviral DNA was 67.4% for R5/R5 and 20.9% for X4/X4 tropisms. This indicated that the Proviral DNA test showed a 90.6% (29/32) of coincidences for R5 tropism and an 81.8% (9/11) for X4 tropism respect to RNA determinated tropism (Table 2). Using FPR cut-off value of 5.75% and single or triplicate testing we obtained a similar number of X4 strains indicating that RNA and proviral DNA tests have similar sensitivity to detect X4 species. In this case, only five discrepancies (11.6%) were detected: 3 were RNA R5/DNA X4 and 2 RNA X4/DNA R5. In a previous work, discrepancies were reported in 4.8% [5]. In order to improve the tropism concordance by RNA and Proviral DNA, we repeated this analysis using 5, 10 and 20% cutoff values as FPR, nevertheless we could not achieve lower values of discrepancies. The comparison between the numbers of discrepancies obtained at each cutoff indicates that the cutoff of 5.75% shows the lowest amount of discrepancies. For instance, the increase in the 10% cutoff eliminates mismatched samples 24 and 37 but produces others in samples 5, 26 and 42 (Table 2). However, with this analysis we can observe a better sensibility to detect X4 variants with a cutoff of 10% and 20%. The major difference to detect X4 variants between RNA and proviral DNA was observed when using FPR cutoff values of 10 and 20% (Additional files 3: Table S3, 4 and 5).

**Table 2:** HIV tropism prediction

**Sample**	**FPR and RNA-based tropism**				**FPR and DNA-based tropism**					
	**FPR1**	**FPR2**	**FPR3**	Tropism*	**FPR1**	**FPR2**	**FPR3**	Tropism*	**Reported results**^**$**^	**Viral load**^**#**^
1	(−)	(−)	0.3	X4	2.4	39.3	31.2	X4	X4	6.95
2	57.1	47.3	57.1	R5	44.2	57.1	57.1	R5	R5	4.90
3	31	31	28.9	R5	28.9	26.2	18.9	R5	R5	3.34
4	1.7	1.7	1.7	X4	0.5	1.7	0.8	X4	X4	4.43
5	(−)	(−)	9	R5	16.7	48.9	35.2	R5	R5	3.83
6	(−)	(−)	24.6	R5	52.2	(−)	89.3	R5	R5	5.72
7	99.4	99.4	99.4	R5	(−)	78.4	99.4	R5	R5	1.28
8	1.7	1.7	1.7	X4	1.7	19.1	1.5	X4	X4	4.04
9	40.1	44.9	44.9	R5	0.5	50.9	0.2	X4	X4	3.38
10	86.5	86.5	86.5	R5	86.5	86.5	86.5	R5	R5	5.11
11	(−)	8.6	5.3	X4	6.8	9.3	5.7	X4	X4	4.58
12	17.2	17.2	39.4	R5	17.2	13.8	17.2	R5	R5	6.59
13	5.4	5.4	4.7	X4	5	5	5	X4	X4	3.91
14	42.6	33.7	42.6	R5	42.6	24.6	24.6	R5	R5	1.28
15	35.1	35.1	35.1	R5	36.9	33.7	35.1	R5	R5	3.61
16	99.4	(−)	99.3	R5	99.9	99.2	99.9	R5	R5	4.54
17	1.7	1.7	1.7	X4	1.1	2.6	1.1	X4	X4	5.67
18	66	33.7	66	R5	(−)	42.2	66	R5	R5	3.66
19	1.9	2.9	2.9	X4	2.9	(−)	0.5	X4	X4	5.96
20	(−)	45.7	45.7	R5	(−)	48.7	(−)	R5	R5	4.41
21	32.2	17.3	15.4	R5	17.9	13.2	19.5	R5	R5	3.26
22	(−)	(−)	6.9	R5	10.6	(−)	6.9	R5	R5	4.34
23	31.4	31.4	31.4	R5	(−)	42.2	(−)	R5	R5	5.36
24	1.7	1.3	(−)	X4	42.2	27.3	6.9	R5	X4	4.86
25	87.1	87.1	87.1	R5	86.2	(−)	(−)	R5	R5	5.73
26	74.4	(−)	(−)	R5	13.8	8.1	37.1	R5	R5	1.28
27	67.3	67.3	22.8	R5	48.9	48.9	(−)	R5	R5	4.65
28	(−)	28.7	(−)	R5	42.2	(−)	12.8	R5	R5	4.23
29	89.1	89.1	89.1	R5	42.2	42.2	(−)	R5	R5	4.18
30	62.5	39.6	48.7	R5	42.2	(−)	(−)	R5	R5	6.28
31	(−)	24.7	(−)	R5	1.7	4.7	42.2	X4	X4	4.04
32	44.6	(−)	44.6	R5	75.6	79.9	0.2	X4	X4	5.88
33	41.6	41.6	41.6	R5	41.6	41.6	41.6	R5	R5	5.26
34	16.6	58.7	58.7	R5	15.4	15.7	(−)	R5	R5	1.28
35	72.3	72.8	72.3	R5	72.3	72.3	(−)	R5	R5	3.23
36	41.6	38.4	(−)	R5	57.6	(−)	(−)	R5	R5	4.30
37	(−)	3.7	(−)	X4	20.4	8.1	8.1	R5	X4	3.78
38	51.2	41.6	51.2	R5	28.5	77.6	45.1	R5	R5	3.38
39	23.1	23.1	23.1	R5	23.1	23.1	23.1	R5	R5	3.20
40	1.7	1.7	2.6	X4	1.7	73.1	73.1	X4	X4	5.46
41	(−)	(−)	0.9	X4	0.2	2.9	23.1	X4	X4	3.08
42	20.9	20.9	20.9	R5	79.9	9.6	(−)	R5	R5	1.28
43	71.5	71.5	71.5	R5	71.5	71.5	71.5	R5	R5	6.59

*Prediction based on the lowest FPR, $ Prediction based on the worst prognosis, #Log (copies/mL), (−) Data not obtained.

On the other hand, through an international collaboration known as external quality assessment (EQA), they established that an adequate performance of DNA-based testing might not tolerate more than two R5 and one X4 discrepancies [15]. The analysis was done with 20 samples, and the discrepancies allowed for R5 and X4 were 10% and 5% of the population respectively. The discrepancies obtained by us for R5 and X4 were 7% and 4.7%. Our results are in agreement with the percentage obtained by EQA and are in concordance with the objectives and results of this international study.

We found that using proviral DNA with a FPR cutoff of 10% and single testing, it was possible to overcome the sensitivity for detection of X4 variants using RNA with a triplicate cutoff of 5.75%. This shows that the increment in the cutoff is useful when the tropism test is performed without replicates. The three FPR values could not be obtained for all the samples. For RNA, three FPR values were obtained in 65%, two in 14% and one in 20.9% of the samples. For proviral DNA, the results were 62.8%, 25.6% and 11.6%, respectively. When retested, these samples did not obtain a V3 loop HIV amplicon or good quality sequences. Similar limitations have been previously reported in 14% of the analyzed samples through proviral DNA and in 10% of samples analyzed through RNA, and failures have not necessarily been related to low viral loads [16]. In our case the explanation for such limitations are poor quality RNA and proviral DNA amplification and sequencing reactions due to primers mismatches, generating low quality sequences that were not approved by RECall. The sequences filtered by Recall were not analyzed manually due to poor quality of chromatograms where we could observe the same results detected previously. For instance, very short sequences, multiple nucleotide ambiguities, or truncated sequences [12]. We will design different primers that will allow improvement of the sequences and we will take more considerations regarding temperature variations when working with RNA. When analyzing FPR values by RNA we found a triple coincidence in 14 samples and a doble coincidence in 15 samples. For proviral DNA we obtained 5 samples with three equal FPR and 9 with two identical FPR values (Table 2). This suggests that the FPR values obtained from RNA have a narrower dispersion than those obtained through proviral DNA with a coefficient of variation (CV) of 0.8995 and 1.0014 respectively. The wider dispersion experienced by proviral DNA might indicate that the cellular fraction has a higher concentration of genetic variants than plasma. This observation had been suggested by other researchers previously [17-20].

We estimated the sensibility of the genotypic method to detect X4 variants using RNA or proviral DNA with single, duplicated or triplicate testing and FPR cutoff values of 5.75, 10 and 20% [16]. We found that the highest sensitivity for detecting the X4 strain occurs with proviral DNA and cutoff of FPR 20% (Additional files 4 and 5). Interestingly, when we used proviral DNA single testing and 10% cutoff, we obtained the same results than using RNA, triplicate and cutoff 5.75%. When we used proviral DNA and single testing with 5.75% cutoff we detected X4 strains with lower sensibility than RNA using single testing and same cutoff (Additional file 4). This result shows the negative impact of the dispersion obtained with proviral DNA tropism and when prediction is performed with single testing and a low FPR cutoff. Our results suggest that is advisable to perform the assay in triplicate when proviral DNA or RNA and FPR cutoff between 5-10% are used. Since triplicate testing increases the likelihood of detecting X4 variants. However, for cases of prediction with one sequence, by RNA or proviral DNA, increasing the FPR cutoff to 20% is recomended [8].

Once the concordance between the RNA and proviral DNA was established, we estimated the prevalence of the X4 variants using a total of 93 samples. We found 30 X4 strain (32.3%), 61 R5 variants (65.6%) and 2 (2.2%) were not reportable. The prevalence found for X4 or R5 strains was similar to previous studies [20]. With a 10 or 20% cutoff, proviral DNA shows a higher sensitivity in detection of X4 species compared to the use of RNA. Therefore, proviral DNA is very useful to determine HIV tropism in cases where low and intermediate viral load cannot be solved with RNA testing [18-20]. Proviral DNA is the only available option for patients with undetectable viral loads requiring a change of therapy [21]. Nevertheless, proviral DNA should be supplemented with deep sequencing to increase X4 minority species detection. Although deep sequencing is not an immediate clinical application, we believe that in a short time this will be possible and this technology will improve the prediction of HIV tropism by genotypic methods [22-24]. Tropism could not be predicted in only two (2.15%) of the samples with undetectable viral loads. This is one of the main advantages of the genotypic method as compared to the phenotypic method in which up to 25% of non-reportable samples have been described [5]. However, a disadvantage of the method based on genotypic plasmatic viral RNA and standard sequencing is that it can only detect variants when these are represented in at least 10% of the population. This limitation has been overcome by using proviral DNA and deep sequencing, as it has allowed more sensitive detection of X4 minority species. Nevertheless, at moment, this strategy still has limited application in routine clinical analysis [22-24]. Alternatively, using standard sequencing, we could monitor the evolution of viral tropism in order to identify when R5-X4 switch occurs. In this way, we can predict the viral tropism in patients simultaneously with the viral load and CD4 counts, so that when we can detect the X4 strains, the physician can change therapy opportunely.

**Figure 2:**
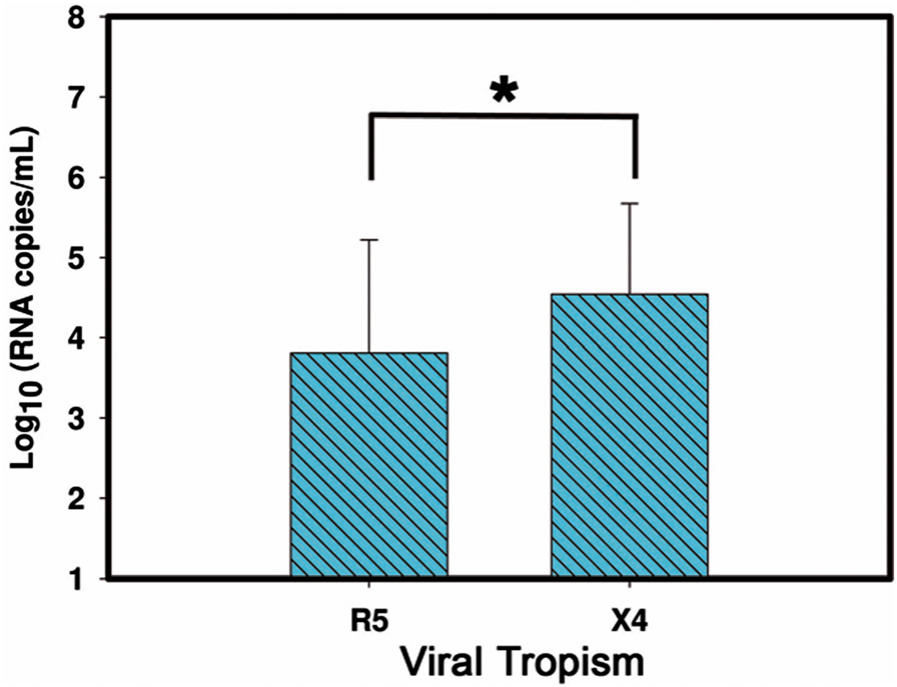
**Relationship between HIV viral tropism and the median of the logarithm of viral load.** The figure shows the median of the viral load of carriers of R5 (n = 61) and X4 (n = 30) strains. The difference in the mean values of the two groups is greater than would be expected by random; there is a statistically significant difference between the input groups (P = 0.017). * = p < 0.05.

When analyzing the possible association between viral tropism and viral load, samples classified as X4 were found to have higher viral loads as compared to R5 samples (P = 0.017) (Figure 2). We found that patients with R5 tropism had higher CD4 counts than patients X4 although the difference was not statistically significant (p > 0.05, Additional file 6). This result together with other previously published, shows that the association between R5 with a lower CD4 cell count was demonstrated, suggesting that detection of X4 variants might be an indicator of poor prognosis for patients recently confirmed with HIV infection [25]. Moreover, it has been observed that X4 HIV, but not R5 HIV, is able to infect hematopoietic stem cells, a fact that may potentially explain low CD4 counts and the poorer prognosis related to X4 strain [26].

The R5 prevalence found in the present work implies that in Chile a high percentage of patients with virologic failure might be eligible for CCR5 antagonist treatment. This information will have an immediate clinical application, since with tropism tests the patients under virological failure in our country may change their therapy. The prevalence reported here was similar to values reported in other studies involving ART-experienced patients [21,27]. Our results corroborate the high concordance in tropism prediction by proviral DNA and by RNA demonstrated previously [5-7]. This work is a contribution in the effort to demonstrate the clinical useful power of the proviral DNA tropism prediction. However, given the wider dispersion of FPR values of proviral DNA found in the present study and demonstrated also by other authors, we consider advisable to continue performing the test in triplicate preferably when FPR 5-10% cutoff values are used [8,15]. When a single sequence is available, the FPR cutoff value should be increased according to European guidelines [8]. Finally, because HIV-1 X4 strains are related to poor prognosis, we consider very appropriate to perform the tropism test in patients recently confirmed with HIV infection together with viral load, CD4 counts and genotyping for traditional antiretroviral drugs.

## Abbreviations

CCR5: The (cysteine-cysteine) chemokine receptor; CXCR4: The (cysteine-X-cysteine) chemokine receptor; HIV: Human immunodeficiency virus; ART: Antiretroviral therapy; RNA: Ribonucleic acid; DNA: Deoxyribonucleic acid; PCR: Polymerase chain reaction; RT: Reverse transcriptase; FPR: False positive ratio.

## Competing interests

The authors declare that they have no competing interests.

## Authors’ contributions

**PF**; participated in the design of the study, performed the RNA and proviral DNA tropisms assays and drafted the manuscript. **LM**; carried out the RNA and proviral DNA tropisms assays. **MT**; performed the statistical analysis and collaborated in the manuscript draft. **RT**; conceived the study, and participated in its design and coordination and collaborated in the manuscript draft. **CR**; conceived the study, and participated in its design and coordination and collaborated in the manuscript draft. **MAG**: conceived the study, and participated in its design and coordination and collaborated in the manuscript draft. **AA**; conceived the study, and participated in its design and coordination and collaborated in the manuscript draft. **MF**; conceived the study, and participated in its design and coordination and collaborated in the manuscript draft. **CB**; conceived the study, and participated in its design and coordination collaborated in the manuscript draft. **CP**; conceived the study, and participated in its design and coordination and collaborated in the manuscript draft. All authors read and approved the final manuscript.

## Supplementary Material

Additional file 1: Table S1 FPR% triplicates, tropism, viral load and viral subtype form 50 additional patients used for estimate the X4 or R5 prevalence. Click here for file

Additional file 2: Table S2 Patient# epidemiological and clinical features of 50 additional samples used for estimate X4 and R5 prevalence. *Median and Range, # All patients with HIV clade B. Click here for file

Additional file 3: Table S3 Tropism predictions based on RNA and proviral using different FPR% cut off. (*) Lower FPR obtained from triplicate analysis. G2P, HIV viral subtype obtained with geno2pheno (coreceptor) bioinformatics tools. Click here for file

Additional file 4 Summary of comparison between RNA and proviral DNA for genotypic prediction tropism, using different FPR% cut off values and single, duplicate or triplicated testing. Click here for file

Additional file 5 Comparison of the sensibility for X4 variants detection by RNA o proviral DNA using different FPR% cut off values. Click here for file

Additional file 6 Relation between X4 or R5 tropism and CD4 counts. Click here for file
