# Supplementary figures and images for: HIV-1 tropism: a comparison between RNA and proviral DNA in routine clinical samples from Chilean patients
Source: Virol J. 2013 Oct 28;10:318. doi: 10.1186/1743-422X-10-318 (PMC4231446; doi:10.1186/1743-422X-10-318)

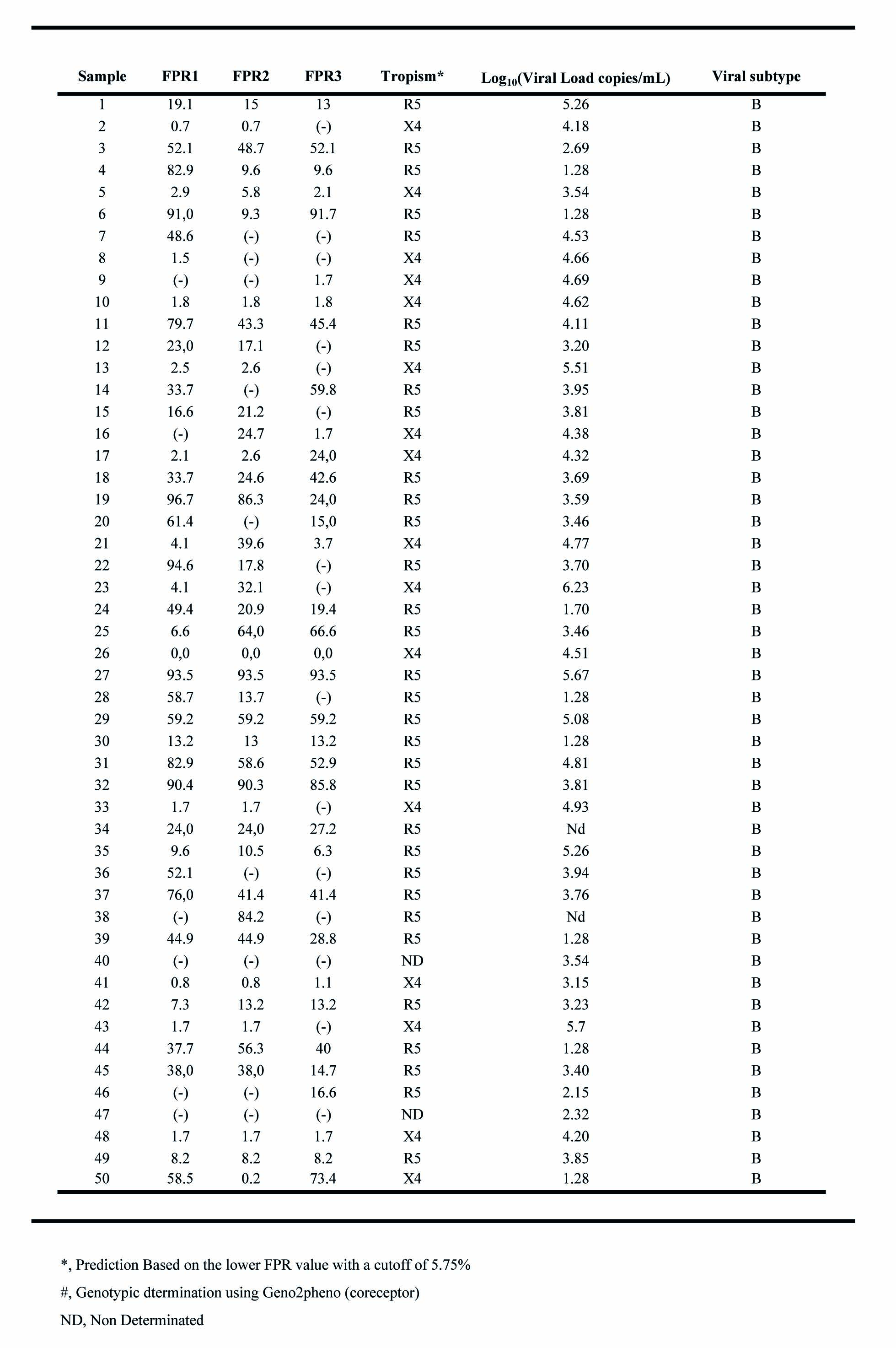

Supplement: Additional file 1: Table S1 — FPR% triplicates, tropism, viral load and viral subtype form 50 additional patients used for estimate the X4 or R5 prevalence. [file 1743-422X-10-318-S1.jpeg]

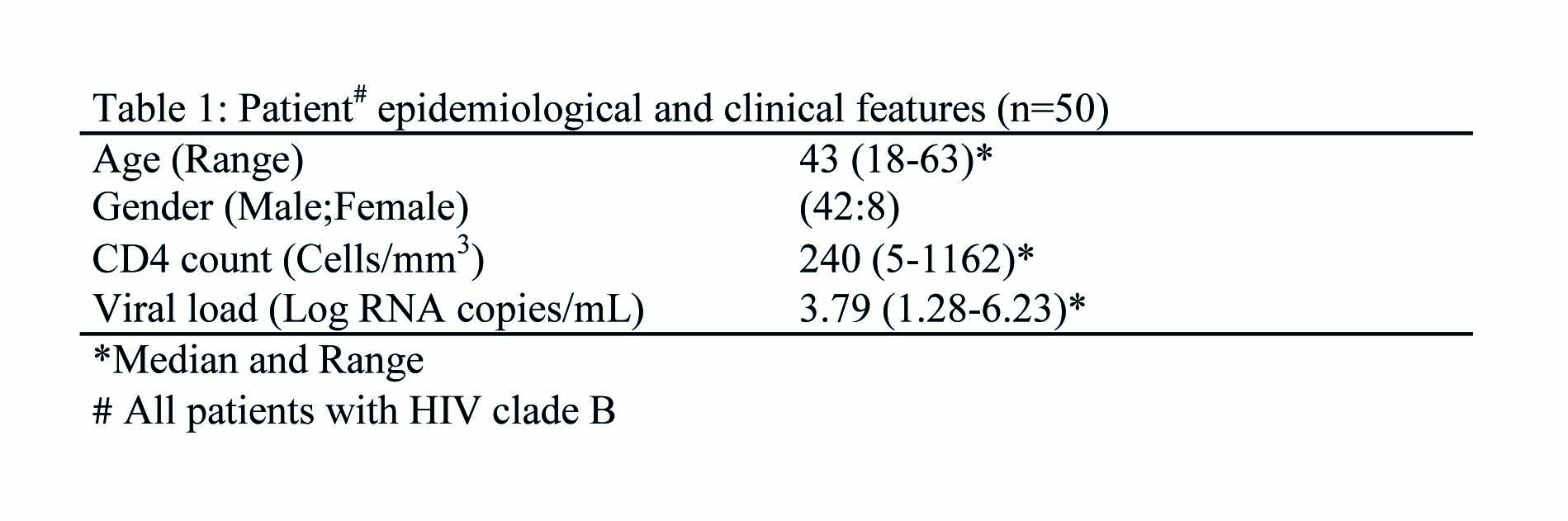

Supplement: Additional file 2: Table S2 — Patient# epidemiological and clinical features of 50 additional samples used for estimate X4 and R5 prevalence. *Median and Range, # All patients with HIV clade B. [file 1743-422X-10-318-S2.jpeg]

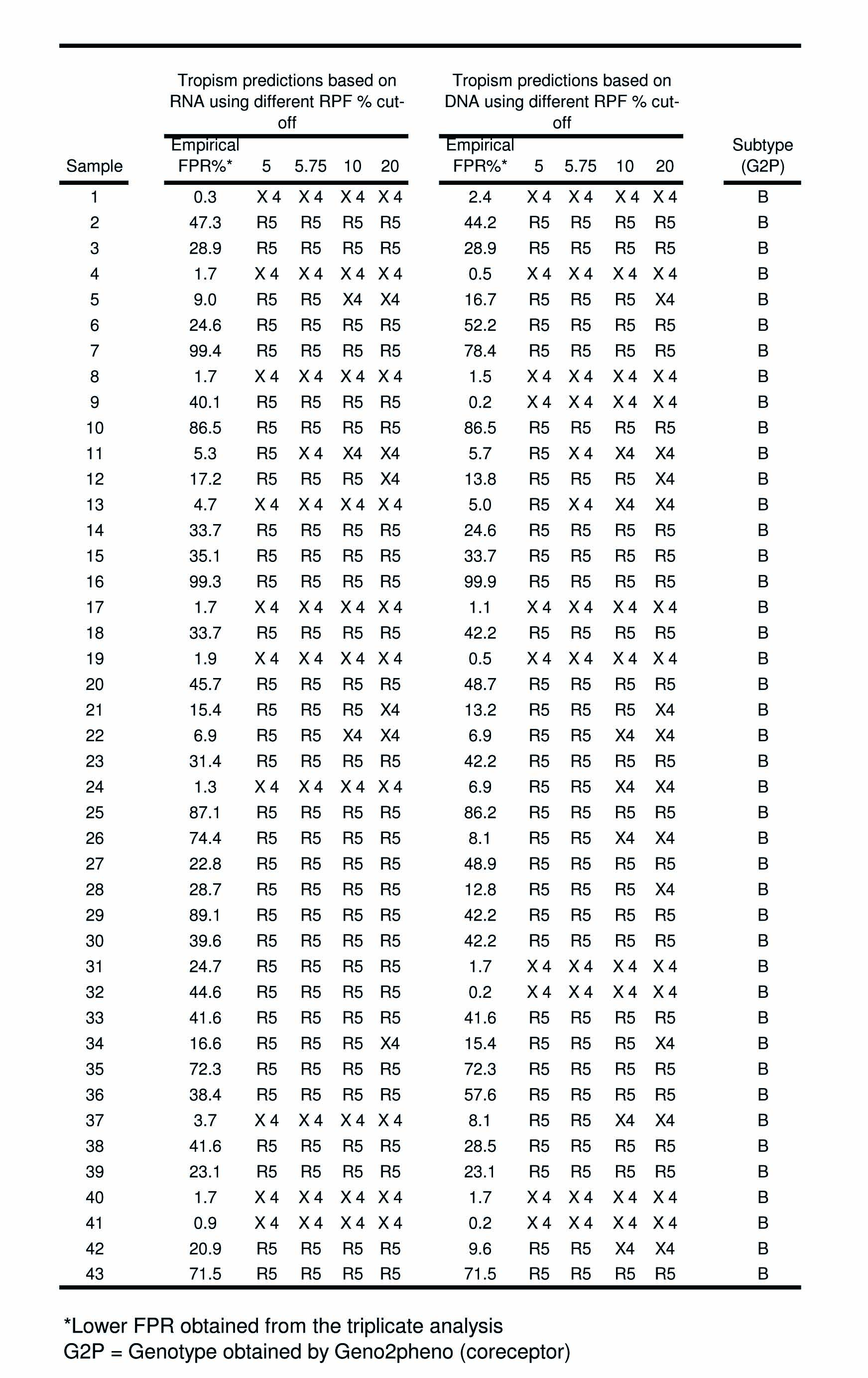

Supplement: Additional file 3: Table S3 — Tropism predictions based on RNA and proviral using different FPR% cut off. (*) Lower FPR obtained from triplicate analysis. G2P, HIV viral subtype obtained with geno2pheno (coreceptor) bioinformatics tools. [file 1743-422X-10-318-S3.jpeg]

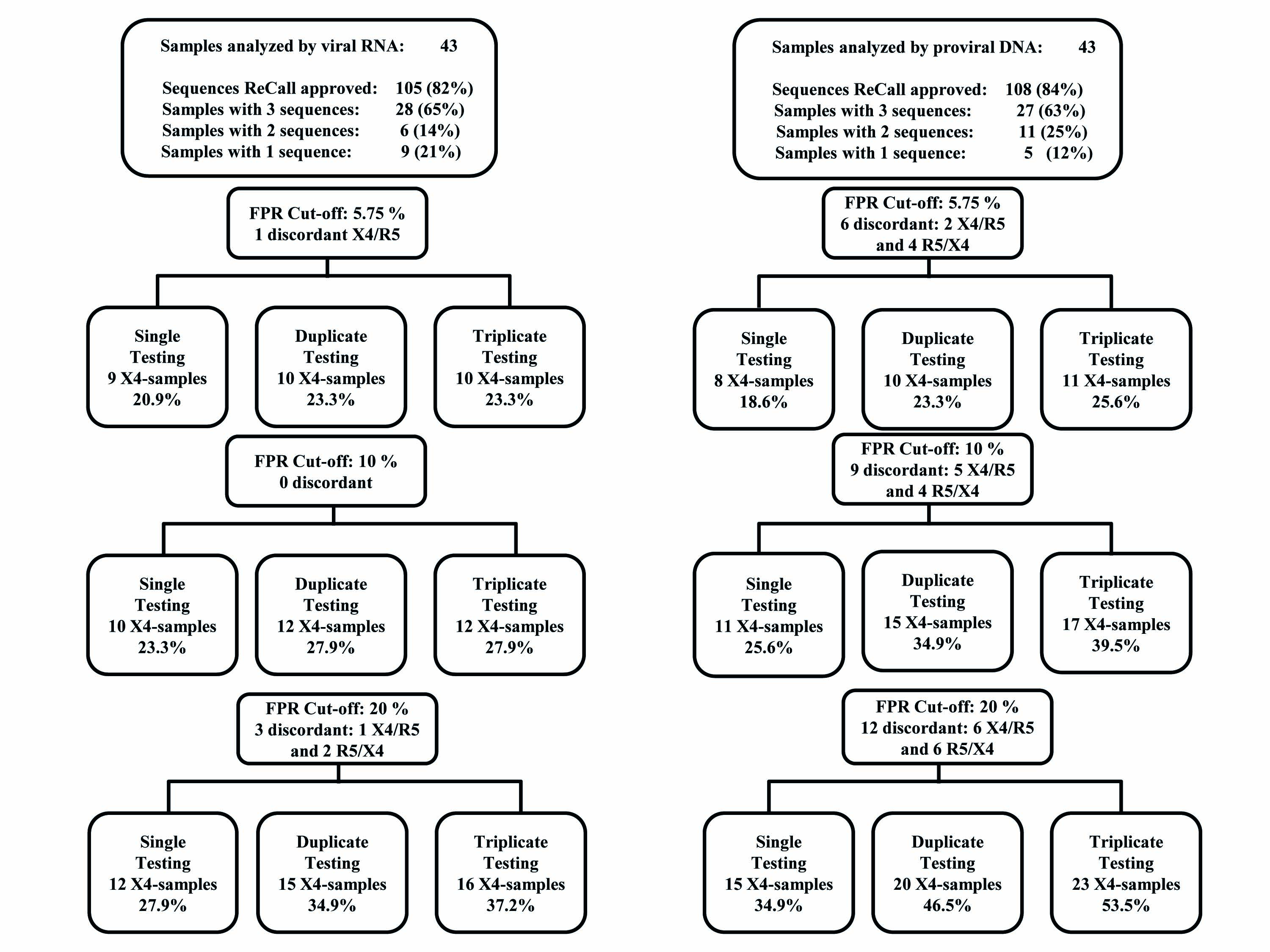

Supplement: Additional file 4 — Summary of comparison between RNA and proviral DNA for genotypic prediction tropism, using different FPR% cut off values and single, duplicate or triplicated testing. [file 1743-422X-10-318-S4.jpeg]

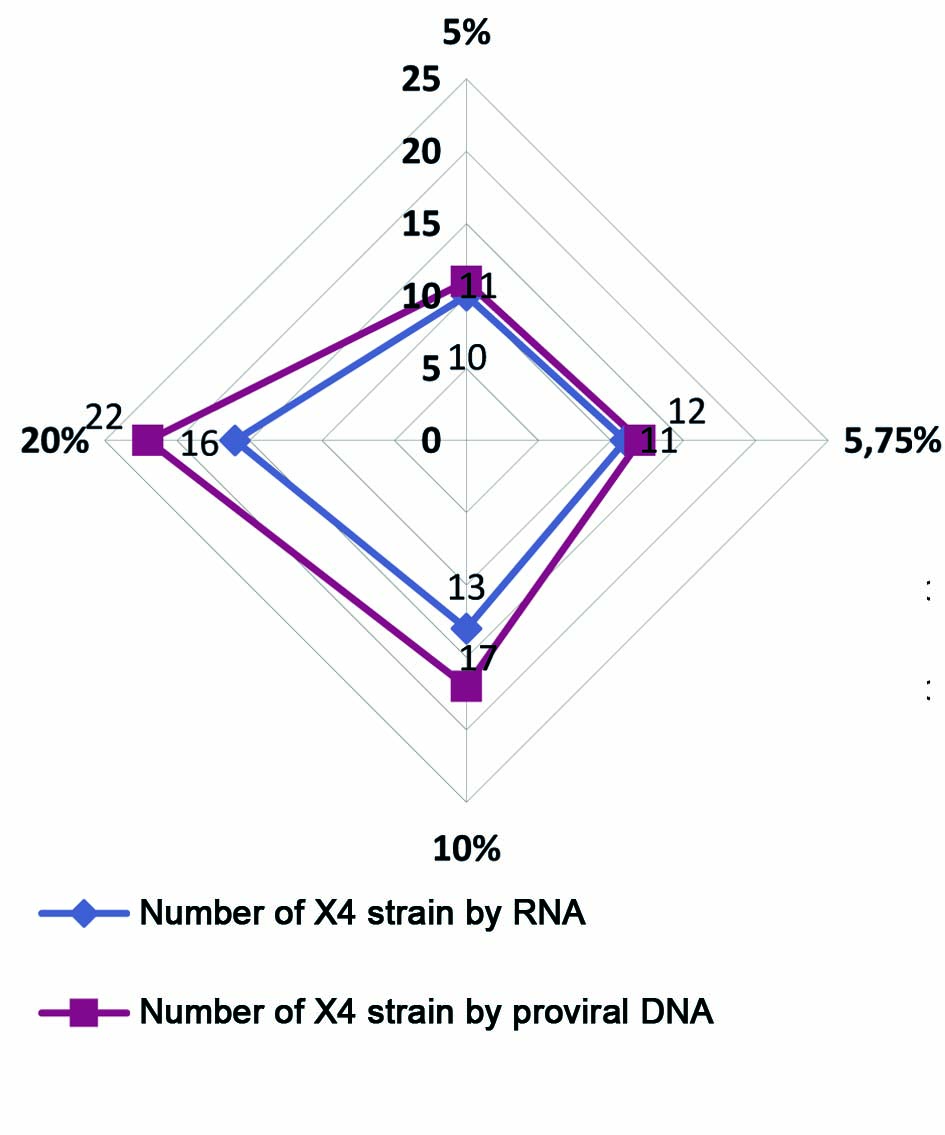

Supplement: Additional file 5 — Comparison of the sensibility for X4 variants detection by RNA o proviral DNA using different FPR% cut off values. [file 1743-422X-10-318-S5.jpeg]

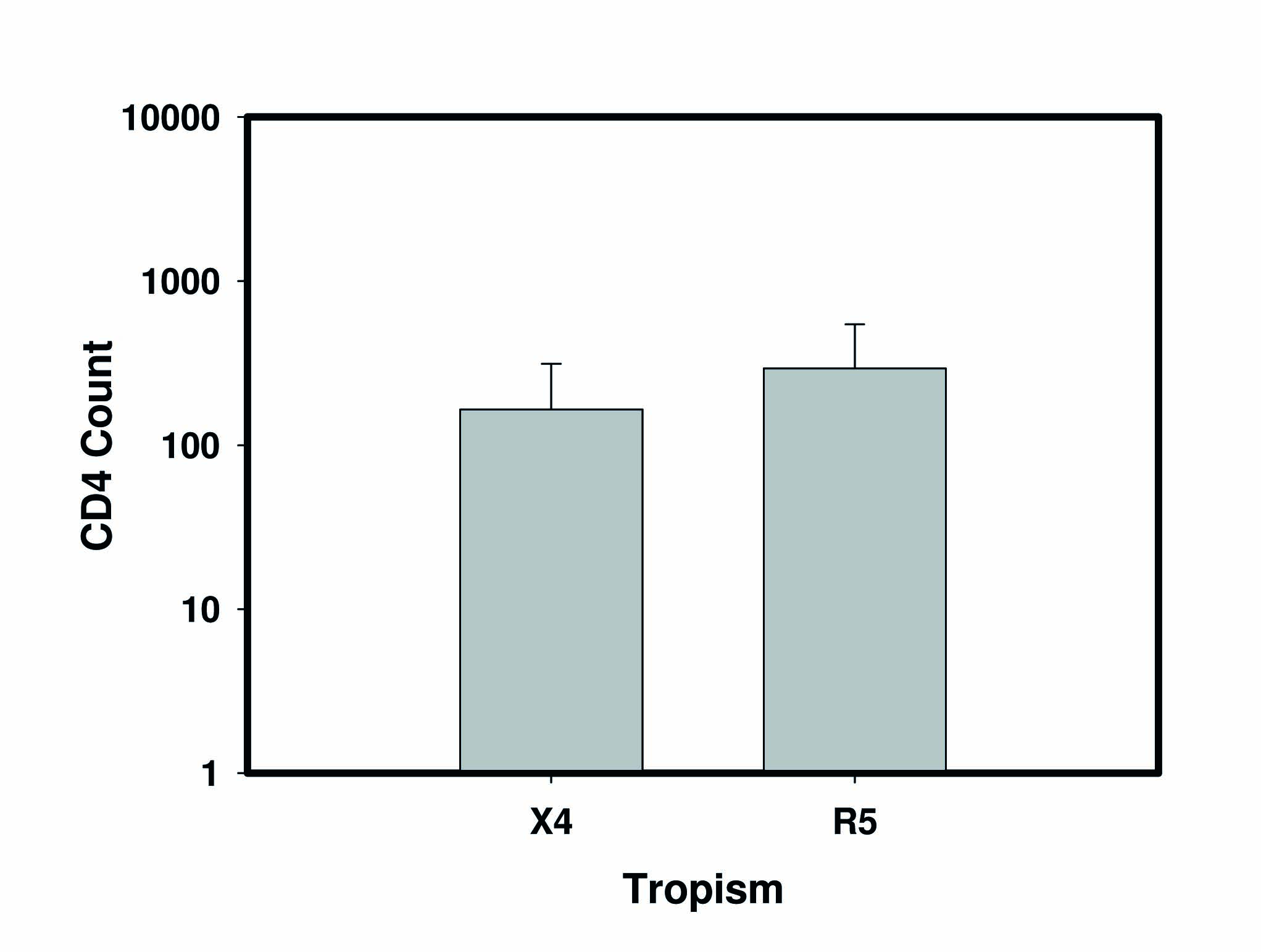

Supplement: Additional file 6 — Relation between X4 or R5 tropism and CD4 counts. [file 1743-422X-10-318-S6.jpeg]
